# SOCIODEMOGRAPHIC DIFFERENCES IN IMMUNOSENESCENCE IN OLDER AGE: EVIDENCE FROM THE HEALTH AND RETIREMENT STUDY

**DOI:** 10.1093/geroni/igac059.1749

**Published:** 2022-12-20

**Authors:** Grace Noppert, Rebecca Stebbins, Jennifer Dowd, Allison Aiello

**Affiliations:** University of Michigan, Ann Arbor, Michigan, United States; King's College London, London, England, United Kingdom; Leverhulme Centre for Demographic Science, University of Oxford, Oxford, England, United Kingdom; Carolina Population Center, University of North Carolina at Chapel Hill, Chapel Hill, North Carolina, United States

## Abstract

Population patterns of immunosenescence are not well described. We characterized markers of immunosenescence and assessed sociodemographic differences in a population of individuals ages 56 years and older using newly released venous blood data from the nationally representative U.S. Health and Retirement Study (HRS) (n=8,400). Median values of the CD8+:CD4+, effector memory (em)RA:naïve CD4+ and emRA:naïve CD8+ T cell ratios were higher among older participants (more aged immune profile) and were lower among those with additional educational attainment (less aged immune profile). Racialized minority populations had immune markers suggestive of a more aged immune profile: Hispanics had a CD8+:CD4+ median value of 0.37 (95% CI: 0.35, 0.39) compared to Whites (0.30, 95% CI: 0.29, 0.31). Blacks had the highest median value of the emRA:naive CD4+ ratio (0.08; 95% CI: 0.07, 0.09) compared to Whites (0.03; 95% CI: 0.028, 0.033). Our regression analyses showed that race/ethnicity and education were associated with large differences in T-cell markers of aging, which were orders of magnitude greater than age. By standardizing regression coefficients to estimate years of immunological aging, we found that each additional level of education was associated with roughly an additional decade of immunological age, and racialized minorities had on average an immunological age two to four decades higher than Whites. As one of the first large-scale population-based investigations of immunosenescence, our study advances understanding of the immune mechanisms underlying age-related disease, with implications for risks such as vulnerability to novel pathogens (e.g., SARS-CoV-2).

